# Understanding preschoolers’ word learning success in different scenarios: disambiguation meets statistical learning and eBook reading

**DOI:** 10.3389/fpsyg.2023.1118142

**Published:** 2023-04-17

**Authors:** Gloria Pino Escobar, Alba Tuninetti, Mark Antoniou, Paola Escudero

**Affiliations:** ^1^The MARCS Institute for Brain, Behaviour and Development, Western Sydney University, Penrith, NSW, Australia; ^2^Australian Research Council Centre of Excellence for the Dynamics of Language, The Australian National University, Canberra, ACT, Australia; ^3^Institute of Cognitive Science, University of Colorado, Boulder, Boulder, CO, United States

**Keywords:** word learning, language acquisition, fast-mapping, disambiguation, mutual exclusivity, statistical learning, storybook, preschool children

## Abstract

Children’s ability to learn new words during their preschool years is crucial for further academic success. Previous research suggests that children rely on different learning mechanisms to acquire new words depending on the available context and linguistic information. To date, there is limited research integrating different paradigms to provide a cohesive view of the mechanisms and processes involved in preschool children’s word learning. We presented 4 year-old children (*n* = 47) with one of three different novel word-learning scenarios to test their ability to connect novel words to their correspondent referents without explicit instruction to do so. The scenarios were tested with three exposure conditions of different nature: (i) mutual exclusivity–target novel word-referent pair presented with a familiar referent, prompting fast-mapping *via* disambiguation, (ii) cross-situational–target novel word-referent pair presented next to an unfamiliar referent prompting statistically tracking the target pairs across trials, and (iii) eBook - target word-referent pairs presented within an audio-visual electronic storybook (eBook), prompting inferring meaning incidentally. Results show children succeed at learning the new words above chance in all three scenarios, with higher performance in eBook and mutual exclusivity than in cross-situational word learning. This illustrates children’s astounding ability to learn while coping with uncertainty and varying degrees of ambiguity, which are common in real-world situations. Findings extend our understanding of how preschoolers learn new words more or less successfully depending on specific word learning scenarios, which should be taken into account when working on vocabulary development for school readiness in the preschool years.

## 1. Introduction

Vocabulary proficiency in preschool children (i.e., 4–6 year-olds) is a strong predictor of school outcomes (Whitehurst and Lonigan, 1998; Uccelli and Páez, 2007; Asaridou et al., 2017), and school success is, in turn, an indicator of positive achievement and earnings later in life (Karoly et al., 2006). A child’s vocabulary depends greatly on their ability to learn new words (Carey and Bartlett, 1978; Sénéchal et al., 1995) and to retain them for later use (Samuelson and McMurray, 2017). Successful word learning implies overcoming different degrees of word ambiguity across contexts (Beck et al., 1983; Hirsh-Pasek et al., 2004; Yu and Smith, 2007). The way children overcome this challenge may depend on the word-learning scenario or condition they are presented with (Samuelson and McMurray, 2017; Byers-Heinlein, 2018), but also on children’s developmental stage (Golinkoff et al., 1994; Hirsh-Pasek et al., 2004) and maturation of their domain-general cognitive processes (Samuelson and Smith, 1998; Vlach and DeBrock, 2017).

Indeed, it has been hypothesized that word learning does not depend on a sole mechanism but on a number of multifaceted linguistic and cognitive processes (Levine et al., 2017). These processes may operate and modulate word learning individually or complementarily in different proportions throughout the lifespan (Golinkoff et al., 1994; Hirsh-Pasek et al., 2004). During the preschool years (i.e., from 3 to 6 years of age), children become experienced word learners, with 6 year-olds acquiring from a few to nearly 20 new words a day (Anglin et al., 1993; Hollich et al., 2000). This impressive word-learning rate results from engaging a combination of processes, mechanisms, and strategies (Hollich et al., 2000; Bion et al., 2013; Levine et al., 2017; Samuelson et al., 2017).

In this study, we consider three common word-learning scenarios that have been widely studied in the language development literature and involve different levels of ambiguity and context for the novel words that are encountered. *Disambiguation*, by applying the mutual exclusivity (ME) assumption (e.g., Horst and Samuelson, 2008), takes place when a novel word and its novel referent are presented alongside one or more familiar referents. *Tracking of statistical co-occurrences via* cross-situational word learning (CSWL) occurs when a novel word is encountered in the context of several possible novel referents without contextual cues to indicate the word-referent pairing (e.g., Smith and Yu, 2008; Suanda et al., 2014; Escudero et al., 2016a). And, *inferring meaning* from storybook narrations (e.g., Senechal and Cornell, 1993; Sénéchal et al., 1995; Ramachandra et al., 2011) where a novel word is accompanied by rich audio-visual contextual cues that aid the connection of word and meaning. While previous studies have examined each scenario separately, we argue that a full grasp of children’s word-learning abilities requires a thorough understanding of the mechanisms involved in each word-learning scenario and a direct comparison of performance across scenarios.

The first step to learning a word is to link a novel label to its referent, an ability referred to as *fast-mapping* (Carey and Bartlett, 1978; Horst and Samuelson, 2008). If a learner is exposed to a novel label-referent association without conflicting information, the novel pair can be fast-mapped effortlessly (i.e., fast mapping one label to one referent). However, if faced with ambiguous information, a disambiguation process helps reduce the number of possible referents for a word (Merriman and Bowman, 1989; Halberda, 2003). In a typical experimental disambiguation task, the learner is exposed to auditory stimuli of a novel label (e.g., where is the *wug*?) and to visual stimuli of a novel referent (e.g., colorful rare object) along with one or more familiar referents (e.g., a ball). To solve the ambiguity, the learner may apply the ME assumption by inferring that *wug* is not the label that corresponds to the referent *ball*. Therefore, the disambiguation effect is a manifestation of ME, and it refers to the learners’ tendency to assign an unfamiliar label to an unfamiliar referent rather than to a familiar referent (Markman and Wachtel, 1988; Merriman and Bowman, 1989; Markman, 1992). Disambiguation occurs in real-life situations when a child is exposed to new or low-frequency words, having already acquired familiar vocabulary items which support the new word-referent (or object) mapping.

Importantly, children apply ME differentially depending on their lexical skills, language experience and cognitive and attentional developmental stage (Davidson et al., 1997; Bion et al., 2013; Kalashnikova et al., 2014). Furthermore, children’s capacity to retain newly learned fast-mapped words over time is not fully understood, with children younger than 36 months not able to retain new ME mappings successfully (Horst and Samuelson, 2008; Bion et al., 2013). Therefore, this word-learning mechanism may be useful for identifying the referent of a novel word in an ambiguous referential situation but does not necessarily lead to long-term retention and learning of the novel word-object association (e.g., Kucker et al., 2015).

In addition to real-life word learning situations involving disambiguation in a single moment of time, there are instances where a child does not have familiar words and referents available to support new mappings but rather a myriad of unknown words and referents. It has been proposed that in these situations, children are able to learn new words *via* CSWL by statistically tracking new labels to referents co-occurring across situations and moments in time (Yu and Smith, 2007). CSWL studies commonly expose the learner to only unfamiliar labels and visual referents without any familiar contextual support (Smith and Yu, 2008; Vlach and DeBrock, 2017, 2019). For instance, in each trial learners are shown two novel referents on the screen, one of them being a target referent (e.g., Object A) and the other a foil referent (e.g., Object B), while simultaneously being exposed to two novel labels (e.g., *dit* and *bon*). The correct association between each label and referent is not apparent until the target label-referent pair (e.g., bon—Object A) appears together in successive trials among various other foils. After being exposed to various labels and referents, the learner can track the statistical information and identify the correct label-referent associations across multiple trials.

Although CSWL abilities have been observed from infancy (Saffran, 2001; Kuhl, 2004; Escudero et al., 2016b), word learning *via* CSWL paradigms improves with age. For instance, while 3 year-olds’ performance appeared to be just above chance (Vlach and DeBrock, 2017, 2019), 5 and 6 year-olds showed better performance in a CSWL paradigm (Vlach and DeBrock, 2017, 2019; Hartley et al., 2020). In addition to age, successful learning depended on the levels of contextual diversity surrounding the target words during the CSWL exposure phase (Suanda et al., 2014). The authors included three variability conditions based on the diversity and frequency with which the foil pairings co-occurred with the non-target words. In the three conditions one foil co-occurred with one target word-referent pairing, however, in the high diversity condition, the target pairings co-occurred with different foils each time, in the mid diversity condition, each foil appeared twice, and in the low diversity condition, each foil appeared three times. Results show that the high diversity condition led to more successful word learning performance compared to the low and mid diversity conditions.

The CSWL paradigm has been used as an intentional (i.e., explicit) task, where the learner is instructed that the task goal is to learn the word-object association, and therefore exposed to a familiarization or task-training phase beforehand (Suanda et al., 2014; Junttila and Ylinen, 2020). However, CSWL has also been presented as an implicit (i.e., unintentional) task, resulting in learners’ rapid inference of novel word-object pairs without training or instructions (Escudero et al., 2016a,c). The absence of explicit instruction creates a level of uncertainty similar to entering a new country or community where a different language is spoken. In this situation, the learner initially understands very little, but after enough exposure they can statistically process speech, segment words and pair words with referents, and eventually learn a new language (see Angwin et al., 2022 for a similar view with adults).

In everyday experiences, children also learn words in rich contexts with many possible referents, which can be mimicked in the lab by exposing learners to storybooks along with narrations (Ramachandra et al., 2011; Abel and Schuele, 2014; Flack and Horst, 2018). Audiobooks accompanied with pictures also lead to word learning in preschoolers, as shown in Ramachandra et al. (2011) who presented an audio-visual storybook containing novel words among novel referents and familiar words. However, a comprehensive meta-analysis of word learning in 2–10 year-old children including published and unpublished studies concluded that the word learning success from storybook reading was highly variable across studies, suggesting a possible publication bias favoring studies with significant effects (Flack et al., 2018). Across studies, children learned around 45% of the words presented to them, and learners’ age did not significantly moderate learning effects, likely because researchers already considered participants’ age when designing each experiment (Flack et al., 2018). The authors concluded that audio-visual narration may promote correct word-referent associations only when certain conditions are appropriate, including the style and context of the narrative (see Horst, 2013; Van den Broek et al., 2018), the number of novel target words, the number of repetitions of the story (see Horst et al., 2011; Read and Quirke, 2018), the characteristics and features of the book (see Strouse et al., 2018), and its illustrations (see also Takacs and Bus, 2018). Importantly, Ramachandra et al. (2011) participants’ word-learning success with an audiovisual book may be due to the narration quality or to the particular characteristics of the word learning paradigm used, namely introducing the novel words one at a time couched in story blocks with five repetitions of the target word. Other studies have shown that children’s word learning is not affected by factors such as type of book, namely printed versus electronic (Gaudreau et al., 2020), or testing modality, namely face-to-face versus online (Escudero et al., under review, 2021). Therefore, success levels may be explained by story content, quantity and quality of the narration, contextual cues and number of target word repetitions (Flack, 2018), which vary across studies making comparisons difficult. Therefore, developing age-appropriate story plots and images may be the key to successful word learning *via* audio-visual books (e.g., Hassinger-Das et al., 2020).

Besides rapid learning of word-object associations, effective word learning involves the ability to retain these associations in time for the novel word to be recognized and retrieved subsequently (Horst and Samuelson, 2008; McMurray et al., 2012; Samuelson and McMurray, 2017). Previous work demonstrates that the degree of ambiguity and difficulty of word-learning scenarios has a direct impact on both immediate and delayed word learning (Vlach and Sandhofer, 2012; Mulak et al., 2019). Particularly, Vlach and Sandhofer (2012) showed that the amount and quality of memory support have an impact on word learning and retention of a new word in 3 year-olds. Children were provided with one, two or three of the following scaffolds to support memory: (i) saliency of the novel referent, (ii) additional repetitions of the new label, and (iii) productive generation of the label. Results showed that the more scaffolds children received, the better word retention they achieved, with scaffolds likely easing the ambiguity in the word learning situation.

At around 4–5 years of age, children get prepared to start formal schooling. During this preschool period, vocabulary size and cognition are critical for school readiness as it predicts academic and cognitive outcomes during the school years (Storch and Whitehurst, 2002; Asaridou et al., 2017). Therefore, a systematic study integrating a variety of learning scenarios is needed to fully understand the mechanisms involved in handling ambiguity and contextual information when recognizing and retaining newly learned words and to further target efforts to help children during this crucial developmental stage. Specifically, a direct comparison of word learning conditions and their delayed retention would contribute to determining the developmental maturation of word-learning strategies in 4 year-old children, with implications for the implementation of efficient word-learning environments in early childhood education.

The present study examines 4 year-old children’s novel word-learning using the three word-learning scenarios reviewed above, namely ME, CSWL and eBook (i.e., an electronic audio-visual storybook) using a methodology that enables direct comparison of word learning accuracy in an immediate and delayed word recognition test. Each word learning condition differs on the exposure characteristics of the target label-referent pairs and the contextual information provided. Specifically, in ME the child is exposed to novel and familiar referents along with novel labels; in CSWL the child is exposed to only novel referents and novel labels; and, in the eBook the child is exposed to novel words and labels couched within narrations and a rich variety of referents. Crucially, the target novel label-referent pairs and the number of exposures to each pair throughout the experiment were identical across the three conditions.

Although we predicted that children would successfully fast-map the novel words under the three word-learning scenarios, we expected children to learn words more successfully when exposed to the ME paradigm compared to the other two scenarios. This is because seeing a familiar object next to the novel object in every learning instance provides an important cue during the fast-mapping process (Lewis et al., 2020), as demonstrated by preschoolers’ high performance in this word-learning scenario in previous studies (Waxman and Booth, 2000; Kalashnikova et al., 2014, 2016). We also predicted that CSWL and eBook word learning would be more challenging than ME because CSWL lacks contextual support, while eBook involves auditory and visual contextual information that may distract young learners. Specifically, CSWL presupposes a heavy memory load for the many occurrences of unfamiliar words and objects across trials, as reflected in learning success just above chance for 3 year-old children (Vlach and DeBrock, 2017, 2019). To learn words from an eBook, children need to select and attend to relevant information (i.e., target new words and objects), while inhibiting potentially more salient irrelevant information (i.e., characteristics of the protagonists, different objects and elements surrounding the depicted scenes, Flack, 2018). Memory contextual support plays an important role in the delayed retention of newly learnt words (Vlach and Sandhofer, 2012), however, we propose that for this to occur, the memory supports should be meaningful and familiar to the learner. Therefore, we predicted that words learned *via* CSWL, due to their lack of contextual support, would exhibit poorer delayed retention than words learned *via* ME and eBook. If the eBook content is meaningful and age-appropriate, delayed retention of learned words would be similar or greater to words learned *via* ME.

## 2. Materials and methods

### 2.1. Participants

Forty-seven children participated in the present study (*M*_age_ = 4.7 years, *SD*_age_ = 0.38 years, *range* = 4.0–5.5 years; 27 females). Children had not started formal school education and did not have a diagnosed language or developmental disorder. Children were recruited from a database of parents who had volunteered to participate in child language research at a university laboratory (*n* = 29) and from childcare centres located in the Greater Sydney area (*n* = 18). Two additional children were recruited but were excluded from the final sample as they refused to start (*n* = 1) or to continue (*n* = 1) the experiment. All children were born in Australia; 28 children (*M*_age_ = 4.7 years, *SD*_age_ = 0.06 years; 16 females) were monolingual speakers of English and 19 children (*M*_age_ = 4.6 years, *SD*_age_ = 0.1 years; 11 females) had additional exposure to another language (*Spanish* = 14, *Mandarin* = 2, *German, Greek and Arabic* = 1 each). To ensure that all children with additional language exposure were proficient in English and understood the English instructions, their English receptive vocabulary was assessed (see in *Receptive vocabulary size* subsection). Children were assigned randomly to one of the three word-learning paradigms: ME (*N* = 15, *M*_age_ = 4.5 years, seven females, five bilingual), CSWL (*N* = 17, *M*_age_ = 4.7 years, seven female, six bilingual), eBook (*N* = 15, *M*_age_ = 4.9 years, eight female, five bilingual). This study was approved by the Western Sydney University Human Research Ethics Committee with number H13141. Participation was voluntary, with parents providing written informed consent and children providing verbal consent before participation.

### 2.2. Materials and apparatus

The experiment consisted of three phases. It began with a learning phase, where one of three different novel word-learning paradigms was administered (i) ME, (ii) CSWL, or (iii) incidental word learning *via* an eBook. An immediate retention test was administered straight after the word-learning paradigm, and a delayed retention test, which was identical to the immediate retention test, was administered 30 min after the novel-word-learning paradigm. There were three familiarization trials in each of the paradigms, for the child to get accustomed to the format of the task, which was administered *via* a touchscreen. The three word-learning paradigms and tests were administered on a Surface Intel Core i5 touchscreen laptop running E-Prime 3 (Psychology Software Tools, 2019). In addition, children completed the toolbox picture vocabulary test (TPVT) as a measure of receptive vocabulary. The TPVT was administered on a 6th-generation iPad. Throughout the experiment, instructions were presented *via* E-Prime 3 along with the stimuli, keeping verbal instructions from the experimenter at a minimum. At the beginning of the experiment, participants were shown both, the touchscreen laptop and the iPad and were told that they would watch some images, hear some words and play some games using the devices. Participants were encouraged to pay attention to the screen but were not instructed to discover which word was associated with which image. Therefore, the three word-exposure scenarios are in essence unintentional or implicit word-learning paradigms.

#### 2.2.1. Target novel words and objects stimuli

**FIGURE 1:**
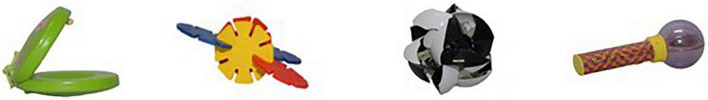
Target novel objects used in the three word learning paradigms.

The novel visual stimuli consisted of four objects (see Figure 1) and the auditory stimuli were four words selected from the Novel Object and Unusual Name-NOUN database (Horst and Hout, 2016). The novel words *wug, lif, pok*, and *neem* have been used in previous research (Byers-Heinlein et al., 2013; Kalashnikova et al., 2018). These words were chosen for their phonetic distinctiveness with no vowel or consonant overlap and their monosyllabic CVC (consonant-vowel-consonant) structure, characteristic of English words (Yap et al., 2015). The target words, the full auditory stimuli (carrier sentences and narrations, depending on the paradigm) and the instructions for all three paradigms were produced and recorded by the same female native speaker of Australian English. Word-object pairings were identical for the three paradigms.

#### 2.2.2. Learning phase: three word-learning tasks

##### 2.2.2.1. Mutual exclusivity (ME)

Prior to the start of the learning phase, children were exposed to three familiarization trials to get used to the touchscreen format of the learning trials. Each familiarization trial consisted of two familiar pictures (an apple, a banana, a chicken, a flower, or a book) and the child was asked to identify one of the familiar objects (i.e., Where is the cat? Find the banana! Touch the book!). The child responded by pressing the corresponding picture on the screen. After this, the mutual exclusivity trials took place as explained below.

**FIGURE 2:**
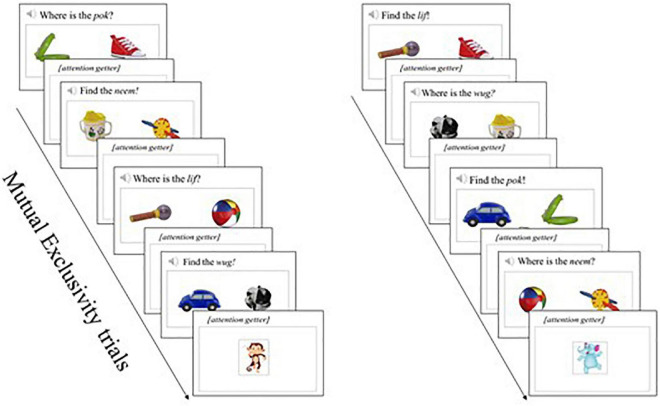
Illustration of mutual exclusivity trials.

In a mutual exclusivity learning trial [adapted from Kalashnikova et al. (2018)], two objects were presented on a white background on a laptop touchscreen, accompanied by an audio recording of a sentence intended to direct the child’s attention to one of the objects. One was the target novel object and the other a familiar object (i.e., an image of a cup, ball, shoe, or car). The auditory stimulus consisted of a carrier sentence containing one of the novel words (i.e., *Where is the [novel word]?; Find the [novel word]*). The carrier sentence lasted for 1,700 ms, and the trial lasted for an additional 3,000 ms where the visual stimuli remained and the child had the option to touch the object on the screen that corresponded to the auditory label (in all cases, this was the novel object). Each learning trial lasted a maximum time of 4,700 ms. If the child touched one of the objects on the screen before the maximum of 4,700 ms, the trial terminated, an attention getter was displayed, and the next trial appeared on the screen. The attention getters consisted of a varied array of child-friendly cartoon images with a synthetic non-word sound and a duration of 1,000 ms each. The attention getters were designed to keep children’s visual attention on the screen. If the child did not choose (i.e., touch) an image, the attention getter was not displayed, and the next trial appeared on the screen. During the learning phase, the child was exposed to 24 learning trials, such that each of the four novel word-object pairs was presented six times in total. Please see Figure 2 for an illustration of ME trials. All participants were exposed to all trials in the same fixed order (Please see Supplementary Table 1 for a detailed list of the ME paradigm word learning trials).

##### 2.2.2.2. Cross situational word learning (CSWL)

**FIGURE 3:**
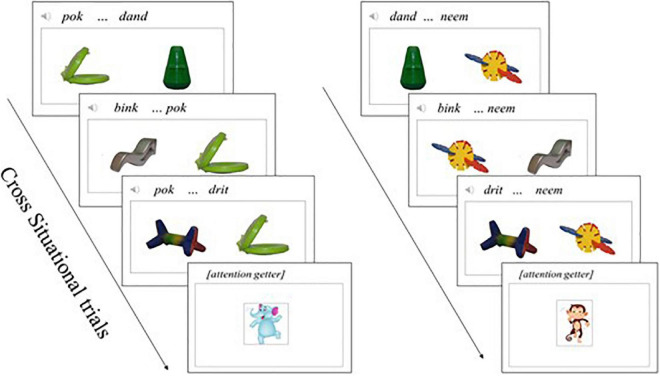
Illustration of cross situational word learning trials.

In a CSWL trial [adapted from Escudero et al. (2016a), Vlach and DeBrock (2017)], two objects were presented on a white background on the laptop screen, accompanied by an audio recording of two isolated words. In each trial the child was exposed to an auditory stimulus consisting of one of the target novel words and one non-target novel word (i.e., *dand, bink, drit, bem, doff, posk*; Horst and Hout, 2016). Each learning trial lasted 3,000 ms, in which a visual stimulus of two figures was presented on the screen; one of them was a target-novel object and the other a non-target-novel object. We created a pseudo-randomized list with the presentation order of the objects (left/right side) and words (first/second) (Suanda et al., 2014). All participants were exposed to all trials in the same fixed order (please see Supplementary Table 1 for a detailed list of the CSWL paradigm word learning trials). During the visual presentation, the recording of two novel words (with a duration of 1,000 ms each, a 500 ms silence between each word and 250 ms of silence at the beginning and end of each word) was presented; the total time of the auditory stimuli plus silence matched the 3,000 ms of the visual stimulus, in line with Vlach and DeBrock (2017). During the learning phase, the child was exposed to 24 learning trials, such that each of the four target novel word-object pairs was presented six times in total. The learning trials were presented in immediate succession, with a blank transition of 250 ms between each of them and an attention getter presented after every three trials, following Vlach and DeBrock’s (2017) arrangement of blocks of learning and attention getter trials. The attention getters were identical to those in the mutual exclusivity paradigm, with a duration of 1,000 ms each. The child was not required to touch the screen at any time during the CSWL learning trials. Figure 3 below shows an illustration of CSWL trials. See Supplementary Table 2 for the CSWL trials’ list.

##### 2.2.2.3. eBook

**FIGURE 4:**
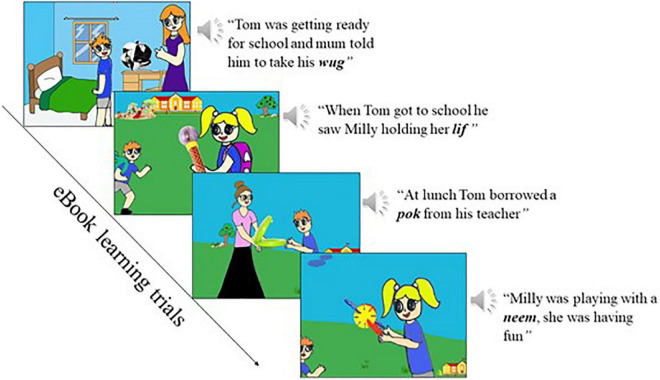
Illustration of eBook trials.

The stimuli consisted of a story presented in an audio-visual eBook format, inspired and adapted from Escudero et al. (under review), Ramachandra et al. (2011). The story was titled “Sharing at School” and consisted of 14 cartoon slides with a pre-recorded audio narrative and visuals. Twelve slides contained the target novel words, while the initial slide was the title page and the last slide was the closing page. The four novel-target words and their visual referents (i.e., novel objects, see section “Target novel words and objects stimuli”) were couched in the story-plot among familiar English words and familiar images depicting a scene. The audio narrative in each cartoon slide lasted 6,000 ms. Then, a red arrow appeared on the inferior-right-hand side of the screen for 3,000 ms while the visual stimuli remained, and the child had the option to touch the arrow to move to the next slide. This red arrow was added in the paradigm to encourage children’s active participation in the story, but this was not a requirement for the task and no data was collected regarding the number of participants that touched the arrow to turn the page. However, observably this option to click to go to the next page of the eBook was hardly used by children because the eBook advanced automatically after the 3,000 ms. During the whole story, children had a total of three exposures to each target novel word and their correspondent object. During the learning phase, the participants listened to the story twice, such that by the end of the eBook learning phase, they were exposed to each novel word-object pair six times. All participants were exposed to all trials in the same fixed order. Figure 4 below illustrates a sample of the eBook pages. Please see the complete eBook story “Sharing at School” with words order list in Supplementary Table 3.

#### 2.2.3. Immediate and delayed retention tests

Prior to the immediate retention test, children participating in the CSWL and eBook conditions were exposed to three familiarization trials to get used to the touchscreen test format and its response method. Each familiarization trial consisted of four pictures in which participants were asked to identify one of four familiar objects (i.e., *Where is the cat? Find the banana! Touch the book!*). The child responded by pressing the corresponding picture on the screen. Children participating in the mutual exclusivity condition were exposed to similar familiarization trials before the learning stage, so this phase was not included for this group.

**FIGURE 5:**
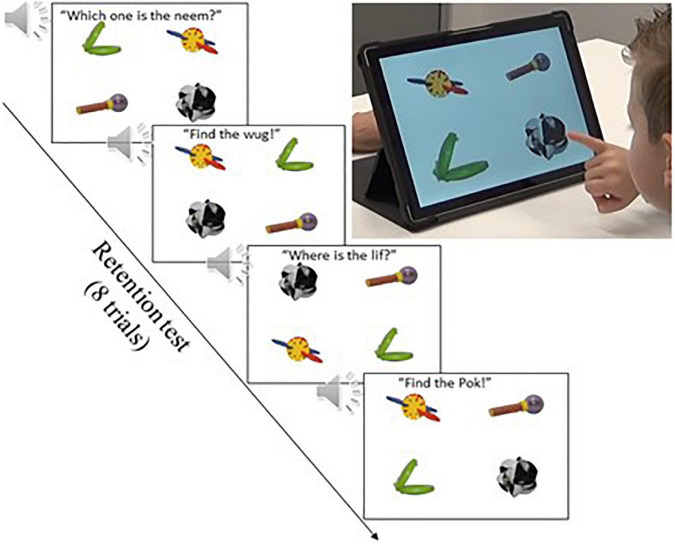
Illustration of the immediate and delayed retention tests.

The immediate and delayed retention tests were identical for all children, regardless of the word-learning paradigm. The retention test was an explicit forced-choice picture recognition test where the participant had to identify the correct object from the four options presented on the screen after hearing its label, as in the familiarization trials. In each test trial, the participant was shown the four target novel objects on the laptop screen and asked to choose the correct one after being asked “Which one is the (novel word)?”. In total there were eight immediate retention test trials. Each of the four novel target words was tested twice. The position of the four novel objects on the screen changed in each trial in a pseudorandom order. See Figure 5 below for an illustration of the retention test trials. Please see Supplementary Table 4 for the full retention test trial list. In both tests, we measured children’s proportions of correct responses (*chance level* = 0.25). Even though we collected children’s reaction times (RT) we only report on their accuracy, as the RT variabilility was high and unreliable. Studies have shown that children’s individual RT strategies and development greatly varies at ages 3–5 (Kiselev et al., 2009; Bucsuházy and Semela, 2017).

After the immediate retention test, the child was administered a receptive vocabulary test and three short screen-based executive function tasks. The three executive function tasks are outside the scope of the present study and will not be reported in the present paper. The child was offered a sticker to attach on a chart as a distraction and reinforcement after completing each task. The total duration of the experimental session was approximately between 35 and 40 min, depending on the child’s disposition. Subsequently, the delayed retention test, which was a repetition of the immediate retention test, was presented as the last activity in the experimental session.

#### 2.2.4. Receptive vocabulary size

*The Toolbox Picture Vocabulary Test—TPVT.* We tested children’s English receptive vocabulary to ensure they understood the task instructions and stimuli. Additionally, evidence shows a relation between vocabulary size and disambiguation trials (Bion et al., 2013), CSWL (Smith and Yu, 2013), and incidental word learning (Sénéchal et al., 1995; Abel and Schuele, 2014). Therefore, it was crucial to take into account participants’ receptive English skills as a predictor of word learning. The TPVT (Gershon et al., 2013) is part of the NIH Toolbox Cognition Battery and is a normed measure of receptive vocabulary for American English, highly compatible with Australian English. All the instructions and procedures of the TPVT were pre-programmed and accessed from the NIH Toolbox App. During the TPVT test, single words were presented *via* an audio file and paired simultaneously with four images of objects, actions, and/or depictions of concepts. The child was asked to select one of the four pictures presented, choosing the one whose meaning aligned best with the spoken word. The selection and number of words administered depended on each participant’s performance in real-time, with a maximum of 25 items. The TPVT was administered on an iPad and took approximately 5 min to administer. The dependent variable for receptive vocabulary was the age-corrected standard score calculated for each participant.

A one-way analysis of variance showed no significant differences in participants’ TPVT scores (*M* = 100.3, *SD* = 12.71) across the three word-learning paradigms *F* (2,44) = 0.499, *p* = 0.61; and a *t*-test revealed no significant difference in TPVT performance between monolingual (*M* = 101.79, *SD* = 14.31) and bilingual children (*M* = 97.95, *SD* = 9.82), *t* (2,44) = 1.01, *p* = 0.31. Indicating that all children had similar English receptive vocabulary skills across word-learning paradigms regardless of their language background.

## 3. Analyses and results

### 3.1. Frequentist analyses

**FIGURE 6:**
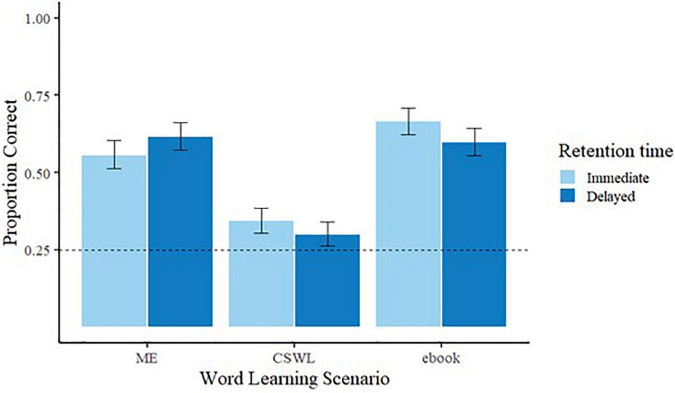
Immediate and delayed retention accuracy across word-learning scenarios (error bars display the standard error of the mean).

Children’s responses from the immediate and delayed retention tests were scored as correct (1) or incorrect (0) and proportions of correct responses were calculated for the analysis. A one-sample *t*-test on the accuracy of children’s responses averaged across all test trials was conducted for each of the word learning scenarios (i.e., ME, CSWL, and eBook) in each of the retention times (i.e., immediate and delayed). Children’s average performance was significantly above chance (0.25) for ME and eBook at both test times (immediate, ME *M* = 0.56 (*SD* = 0.26), *t* (14) = 4.63, *p* < 0.001, and eBook *M* = 0.66 (*SD* = 0.24), *t* (14) = 6.61, *p* < 0.001 and delayed, ME *M* = 0.62 (*SD* = 0.25), *t* (14) = 5.63, *p* < 0.001, eBook *M* = 0.60 (*SD* = 0.22), *t* (14) = 6.23, *p* < 0.001). In contrast, CSWL was nearly above chance for immediate *M* = 0.35 (*SD* = 0.23), *t* (16) = 1.70, *p* = 0.05 but not above chance for delayed retention, *M* = 0.30 (*SD* = 0.21), *t* (16) = 1.00, *p* = 0.17. A Bonferroni corrected alpha level (three comparisons) of 0.016 more clearly shows that CSWL cannot be regarded as different from chance for either immediate or delayed retention^1^ (see Figure 6 for a graphic representation of the immediate and delayed retention accuracy across word-learning scenarios).

We then conducted a series of logistic binomial Generalized Linear Mixed Models using the glmer function from the lme4 package (Bates et al., 2015) in R (RStudio Team, 2020) to compare word-learning accuracy across the three word learning scenarios, revealing a significant effect of Word Learning Paradigm but no effects of Time (i.e., immediate vs. delayed retention time) or receptive vocabulary, as measured by the TPVT^2^. Although these results seemed compelling we decided to conduct a Bayesian analysis to explore the level of certainty for our results and to include factors such as language background (i.e., bilingual vs. monolingual) that had not been included due to the limited number of participants.

### 3.2. Bayesian regression modeling

To analyze the relationships between predictors (i.e., word learning paradigm, English receptive vocabulary as per TPVT, language background, and retention time) and outcome (i.e., response accuracy), we conducted multilevel Bayesian regression modeling using the brms package from the statistical program R using Stan (Bürkner, 2017, 2018; RStudio Team, 2020). We chose this particular analysis method because it provides a number of advantages over the classical frequentist statistical approach. The Bayesian approach is particularly suited for the type of data from this study as it allows for the estimation of complex models where frequentist methods do not provide robust results or are not appropriate. Noisy child data are a typical example of complex data sets, with small or unbalanced samples, which yield low statistical power using conventional statistics (Van de Schoot et al., 2017, 2021; Escudero et al., 2020). A similar Bayesian approach has been used in many recent papers to handle complex data sets (e.g., Smit et al., 2019, 2022; Escudero et al., 2020, 2022a,2022b; Milne and Herff, 2020).

The multilevel (also known as hierarchical) Bayesian mixed regression model is the Bayesian analog of a frequentist generalized mixed effects model as it contains population-level effects (i.e., similar to fixed effects) and group-level effects (i.e., similar to random effects) (Milne and Herff, 2020). In the present study, we have three such “group-level”: “participants,” “trials” (i.e., eight testing trials) and “time” (i.e., retention times, immediate and delayed), hence the population-level effects are the estimated means of the effects across all participants, trials and times tested. The group-level effects (standard deviations and correlations) estimate covariations from the mean across participants, and across trials and retention times (beyond that accounted for by the model’s fixed effects for each paradigm condition, which are ME, CSWL, eBook, and their interactions).

Similar to the Bayesian analysis conducted in Milne and Herff (2020), our modeling uses a maximal group-level (random) effects structure; that is, we allow all within-participant variables to vary by participant, and all within-condition variables to vary by each paradigm condition. These maximal random effects structures often fail to converge using frequentist analyses but are feasible with Bayesian regressions. Furthermore, this analysis calculates the whole posterior probability distribution of each effect rather than only a point-estimate of the most probable effect of each predictor. We can then calculate credibility intervals (instead of confidence intervals from the frequentist approach) and evidence ratios for a given effect to be greater than 0. Since *p*-values are not calculated in Bayesian modeling, the uncertainty of the significant/non-significant dichotomy and null effect interpretations from the frequentist approach are overcome by focusing on probability, effect sizes and evidence ratios in a continuum (Escudero et al., under review). A 95% credibility interval is analogous to a significant *p*-value. If the (two-sided) 95% credibility interval does not contain 0, less than 2.5% of the posterior distribution is located on the other side of 0. A hypothesis-testing method determines whether one parameter’s interval differs from another by calculating evidence ratios and therefore quantifying the likelihood of a given hypothesis with respect to the alternative. Evidence ratios above 30 represent “very strong” evidence for a given hypothesis (Escudero et al., 2020) and evidence ratios of 19 are roughly similar to “significant” at an α = 0.05 level (Milne and Herff, 2020).

Default weakly informative priors were used on this Bayesian approach. We used a prior with a Student’s *t*-distribution with 3^°^ of freedom, a mean of 0, and a scale of 1. The TPVT score, being a non-binary predictor was standardized with 3^°^ of freedom, a mean of 0, and a standard deviation (SD) of 1. We used visual exploration and approximate leave-one-out (LOO) cross validation to find the optimal model which assigns the highest probability, avoids overfitting and explains the data. In this way, we ensured obtaining the model that includes only the relevant factors that best explain our dependent variable or outcome (i.e., response accuracy) within and across the three conditions (i.e., ME, CSW, and eBook).

### 3.3. Bayesian modeling results

**FIGURE 7:**
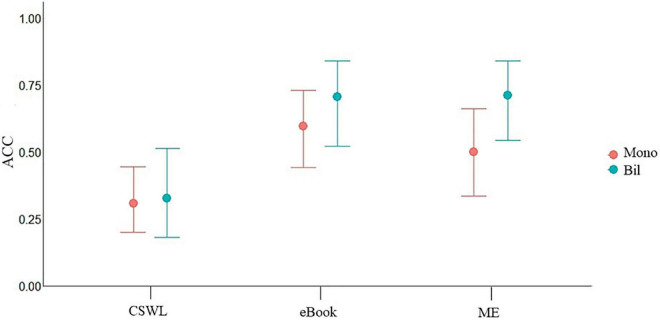
Visualization of “Condition*lgroup summed Model”. Lgroup_sum includes Mono, monolingual; Bil, bilingual; ACC, response accuracy.

We first explored whether terms for Trial, Time (i.e., *IRet* = immediate vs. *DRet* = delayed), TPVT (i.e., receptive vocabulary), language background (i.e., lgroup) or their interactions should be included to test effects between paradigm conditions (i.e., ME, CSWL, and eBook) response accuracy (i.e., ACC). Initial models revealed that Trial, Time (i.e., immediate vs. delayed) and TPVT were not meaningful factors for explaining the dependent variable (i.e., ACC) but condition and language background were. This led us to a model using sum contrasts of interactions of both factors condition and language background (using the named_contr_sum function in R). Sum contrasts are values assigned to predictor variables to encode specific comparisons between factor levels and to create predictor terms to estimate these comparisons (Nicenboim et al., 2022). With sum contrasts, the intercept maintains the interpretation of the corrected mean and therefore performing a summed model ensured that all of the effects are balanced around the intercept, which is the overall mean. The model that most accurately revealed the probability of achieving a certain accuracy given the meaningful variables was “Condition*lgroup summed Model” and was obtained with the formula: ACC∼1 + Condition_sum*lgroup_sum + (1 | Participant). See Table 1 for the output as per standard notation used in R and Figure 7 for visualization of the model.

**TABLE 1:** “Condition*lgroup summed Model” as per standard notation used in R.

Family	Links	Formula	Data	Draws			
Bernoulli	mu = logit	ACC∼1 + Condition_sum* lgroup_ sum + (1 | Participant)	Data (Number of observations: 752)	Four chains, each with iter = 2,000; warmup = 1,000; thin = 1 Total post-warmup draws = 4,000			
**Group-level effects**							
**∼Participant (number of levels: 47)**							
	**Estimate**	**Est. error**	**l–95% CI**	**u–95% CI**	**Rhat**	**Bulk_ESS**	**Tail_ESS**
sd (Intercept)	0.81	0.15	0.55	1.12	1.00	1,720	2,442
**Population-level effects**							
	**Estimate**	**Est. error**	**l–95% CI**	**u–95% CI**	**Rhat**	**–**	**–**
Intercept	0.11	0.14	−0.17	0.39	1.00	–	–
Condition_CSWL	−0.87	0.2.	−1.27	−0.46	1.00	–	–
Condition_ebook	0.52	0.21	0.11	0.93	1.00	–	–
lgroup_ Mono	−0.25	0.15	−0.54	0.04	1.00	–	–
Condition_CSWL: lgroup_Mono	0.20	0.21	−0.20	0.61	1.00	–	–
Condition_ebook: lgroup_Mono	0.00	0.21	−0.40	0.40	1.00	–	–

Estimate, estimated mean of the standardized effect; Est. error, standard error; l–95% CI u–95% CI = 95% credibility interval. Rhat, R-hat convergence diagnostic. Bulk_ESS, bulk effective sample size; Tail_ESS, tail effective sample size.

The model converged well as diagnosed by Rhat with values of one, meaning that the within and between factors’ chains were appropriately mixed as sufficient iterations were produced and the chosen priors were robust enough (Bürkner, 2017). As shown in Table 1, the term for Condition ME is hidden, as it was not shown in the results’ output. Therefore, to model the hypotheses, a logic was inferred where *ME* = –(CSWL + eBook) hence the hypotheses including ME contain simplified equations derived from it (e.g., CSWL–*ME* = CSWL– –(CSWL + Book) = CSWL + CSWL + Book = 2*CSWL + Book). With this method, we revealed the hidden terms in the model’s output, similar to performing an ANOVA to a generalized linear mixed model but in probabilistic terms. Table 2 below shows a summary of directional hypotheses.

**TABLE 2:** Summary of directional hypotheses tests for the effects in the Condition*lgroup summed Model as per standard notation used in R.

Hypothesis	Estimate	Est. error	95% CI		Evid. ratio	Post. prob	Star
CSWL—eBook (CSWL—eBook < 0)	−1.39	0.35	−1.97	−0.8	3,999	1	*
CSWL—ME (2*CSWL + eBook < 0)	−1.22	0.36	−1.81	−0.64	1332.33	1	*
eBook—ME (2*eBook + CSWL < 0)	0.18	0.36	−0.42	0.75	0.45	0.31	–
Monolingual–Bilingual (lgroup-Mono < 0)	−0.25	0.15	−0.49	0	20.39	0.95	*

The columns from left to right are: the hypothesis being tested; the estimated mean of the standardized effect; standard error; 95% credibility interval; the evidence ratio in favor of the hypothesis; the posterior probability for the hypothesis; a star, if zero lies outside the 95% CI.

As shown in Table 2 above, the probability of performance differences between CSWL and eBook is “very strong” with an evidence ratio of 3,999 showing a better performance when exposed to eBook than CSWL. The probability of difference between CSWL and ME is also “very strong” with an evidence ratio of 1332.33 showing a better performance when exposed to ME than CSWL. There is a minimal probability of performance difference between eBook and ME as 0 lies inside the 95% CI and the evidence ratio is less than one. Finally, there is a weak to moderate probability of performance difference between monolingual and bilingual participants, indicating lower accuracy for monolinguals in comparison with bilinguals, as illustrated in Figure 7.

## 4. Discussion

The present study is the first to systematically test three different word-learning paradigms in one study. It integrates the initial mapping stage with immediate and delayed retention tests as crucial indicators of word learning under three different word exposure conditions. Preschool children were presented with six repetitions of the same novel words and referents *via* either ME, CSWL, or the eBook paradigm. We predicted that children would be able to learn the words from the three paradigms, but that they would perform better in the ME paradigm compared to the CSWL and eBook. We expected that the familiar referent in each ME trial would aid the fast-mapping process as it has been shown that children prefer to assign an unfamiliar label to an unfamiliar referent. Furthermore, we predicted that the high memory load required in the CSWL paradigm would tax participants’ performance, and that the colorful visual referents and abundant phrases in the eBook would potentially distract children from linking the target word-referent pairings. We used Bayesian modeling to harness robust results and observations despite sample sizes. Our predictions were partially confirmed as children learned the novel words better in the ME and eBook paradigms compared to the CSWL paradigm. Children’s high accuracy in the ME condition is in line with previous findings (Holland et al., 2015; Lewis et al., 2020), while low CSWL performance, with delayed retention at chance, supports Vlach and DeBrock (2017, 2019) findings. Contrary to our predictions, children were highly successful at learning the target words with the eBook, suggesting that the visual and auditory elements in the eBook were appropriate for the developmental stage of the participants (Flack et al., 2018).

The word learning strategies employed in our ME and CSWL conditions are essential for establishing accurate links between a novel word and its referent, however, children’s daily word learning experiences are very complex (Levine et al., 2017; Samuelson and McMurray, 2017; Vlach and DeBrock, 2017) and require a combination of learning strategies to successfully learn new vocabulary. From a young age, children learn words through the confluence of personal, and often conscious, engagement, effort, and emotion along with the involvement of cognitive and affective processes (Bloom, 2000). The eBook provided the opportunity to apply multiple learning strategies, such as ME for novel object-word pairs among familiar referents and CSWL to establish the correct pairing of novel words across scenes. Importantly, the eBook provided engagement and motivation to deploy the attentional and memory processes necessary to achieve successful word learning. As confirmed by a follow-up study, high enjoyment was reported from participating in the eBook paradigm, even when administered online (Escudero et al., under review), suggesting that participants face-to-face may have been highly engaged as well. Due to the diversity of studies and methods employed (Wasik et al., 2016; Flack et al., 2018), story time effects on word learning had been difficult to assess. Our findings confirm that the richness of audio-visual and contextual cues surrounding a novel word, rather than isolated words and meanings, increases the learner’s engagement and attunes attentional and cognitive resources optimally, in line with Suanda et al. (2014) findings that more contextual diversity is beneficial for young children. Although memory processes have been long deemed as crucial for word retention (Alloway et al., 2004; Gathercole et al., 2004; Vlach and Sandhofer, 2012; Vlach and DeBrock, 2017), our findings suggest that attentional processes and learner’s engagement are as important as memory skills for preschoolers’ word learning.

Unlike previous studies showing that 2 year-old children forget fast-mapped words after 5 min (Horst and Samuelson, 2008), the 4 year-old children in the present study remembered the learned words 35 min later. This demonstrates a substantial increase in word learning and retention by 4 years of age. However, the CSWL paradigm yielded the least efficient retention, likely because it presented the highest memory load with only novel referents that resulted in high ambiguity. This observation aligns with previous adult studies that have pointed out that degrees of ambiguity (i.e., *CSWL* = highest ambiguity, *ME* = lowest ambiguity out of the three paradigms) and difficulty of word-learning scenarios have a direct impact on both immediate word learning outcomes and retention outcomes over time (Vlach and Sandhofer, 2012, 2014; Mulak et al., 2019). Our results therefore extend those of Vlach and Sandhofer (2012) in a conclusive way, as the present study was conducted with four novel words instead of one.

Against our predictions, results of the receptive vocabulary test did not predict word learning, despite previous studies showing that vocabulary proficiency predicts word learning from narratives (Sénéchal et al., 1995; Abel and Schuele, 2014) and novel word retention (Kucker and Samuelson, 2012; Bion et al., 2013; Samuelson et al., 2017). The small variability in the test scores for children in the present study may have contributed to the lack of effect of vocabulary proficiency. Crucially, our results align with previous findings showing that for preschoolers (Vlach and DeBrock, 2019; Hartley et al., 2020) and 2 year-olds (Kucker et al., 2020) CSWL was not predicted by vocabulary size. Our results may indicate that children’s lexical reliance on disambiguation may have shifted to more complex language elements or structural information not present in our paradigms, such as word order and grammaticality (Yoshida and Hanania, 2013). Therefore, at least for the paradigms we presented, children may rely more on cognitive resources than on their lexical knowledge, in line with findings that word learning emerges from domain-general cognitive processes (Samuelson and Smith, 1998; Samuelson and McMurray, 2017; Vlach and DeBrock, 2017).

Interestingly, we were not able to find a compelling effect of bilingualism. Although the summed model suggested an effect of bilingualism on ME, the moderate evidence ratio of 20.39 does not allow for conclusive interpretations. As mentioned above, an evidence ratio of 19 is roughly equivalent to a *p*-value of 0.05 on frequentist statistics (Milne and Herff, 2020), which could be considered disputable evidence to reject the null hypothesis. Our results are counter to previous studies finding less reliance on ME for bilingual children, as they know that one object can have two or more translational equivalents (Kalashnikova et al., 2015). A unique feature of the present study is that monolingual and bilingual children had comparable receptive vocabulary scores, while in most previous studies including Kalashnikova et al. (2015), monolingual children had higher receptive vocabulary scores than their bilingual peers (see also Hoff, 2013; Hoff and Core, 2013). Additionally, considering that Yoshida et al. (2011) found a bilingual advantage for novel adjective learning related to inhibition, which aligns with previous studies showing a bilingual advantage for complex aspects of inhibition (i.e., interference suppression, Pino Escobar et al., 2018), it is plausible that our experiments did not tap on this specific cognitive resource in bilinguals. Follow-up studies should include bilingual children with more variable vocabulary proficiency and complex paradigms to tap on inhibition.

The present study demonstrates that 4 year-old children are successful at learning words across three word-learning scenarios: ME, CSWL, and an eBook. Crucially, different word-learning scenarios foster different learning outcomes, with eBook and disambiguation *via* ME facilitating rapid and more accurate word learning than CSWL. Children may initially acquire a limited number of vocabulary items and when enough familiar words are found in their repertoire, disambiguating through the ME assumption may occur. Exposure to rich environments such as books or eBooks may help initial learning, while statistical abilities to learn words may be used with time. Therefore, the contextual information and referential input provided by the ME assumption and the story of an eBook should be considered in early childhood education settings to support lexical development, especially for children with small vocabularies.

## Data availability statement

The raw data supporting the conclusions of this article will be made available by the authors, without undue reservation.

## Ethics statement

The studies involving human participants were reviewed and approved by Western Sydney University Human Research Ethics Committee with approval number H13141. Written informed consent to participate in this study was provided by the participants’ legal guardian/next of kin.

## Author contributions

GP, PE, AT, and MA contributed to the conception and design of the study. GP collected the data, organized the database, wrote the first draft of the manuscript, and performed the statistical analyses with contributions from AT and PE. All authors contributed to manuscript revision, read, and approved the submitted version.

## Footnotes

1 As suggested by a reviewer, we added Bonferroni correction to our analysis. However, we would like to acknowledge that this type of correction is highly conservative and may lead to high rate of false negatives. Therefore, it should only be used when several multiple tests are involved and when tested are not independent of each other (Brown, 2008; Armstrong, 2014).

2 We started with a model (Model 1) including Word Learning Paradigm (i.e., ME, CSWL, eBook) as a fixed effect and intercepts for Participant and Trial. The model revealed a significant effect of Word Learning Paradigm, *F* (2,45) = 11.31, *p* < 0.001, *AIC* = 949.3. A *post-hoc* Tukey test showed that children learned more word-referent associations from the eBook than from CSWL (*p* < 0.001); more from ME than from CSWL (*p* < 0.001), and there was no difference between the eBook and ME (*p* = 0.82). We then included Time (i.e., immediate and delayed retention) as a fixed effect in a subsequent model (Model 2). The fixed effect of Time (*p* = 0.57, *AIC* = 951.0) was not significant and did not improve the predictive fit of the model, as evidenced by a higher AIC value, indicating that children were able to retain the newly learnt words to the same extent immediately after the learning phase and 35 min later. Adding the Word Learning Paradigm*Time interaction (*p* = 0.40, *AIC* = 952.3) or TPVT (*p* = 0.99, *AIC* = 953) as fixed effects to Model 1 also did not improve Model fit, suggesting that retention did not depend on the word learning paradigm, and it was not influenced by receptive vocabulary.
