# Supplementary material for: Understanding preschoolers’ word learning success in different scenarios: disambiguation meets statistical learning and eBook reading
Source: Front Psychol. 2023 Apr 17;14:1118142. doi: 10.3389/fpsyg.2023.1118142 (PMC10150025; doi:10.3389/fpsyg.2023.1118142)
Supplement: Supplementary file 1 [file Data_Sheet_1.docx]

Supplementary Material

Understanding preschoolers’ word-learning success in different scenarios: Disambiguation meets statistical learning and eBook reading

**Gloria Pino Escobar*, Alba Tuninetti, Mark Antoniou & Paola Escudero**

*** Correspondence:** Corresponding Author: g.pinoescobar@westernsydney.edu.au

**Supplementary Table 1. Mutual Exclusivity (ME) paradigm word learning trials’ list.** **Stimuli objects are represented in capital letters.**

| Mutual Exclusivity (ME) paradigm word learning trials’ list. | | |
| --- | --- | --- |
| Picture 1 | Picture 2 | Sound |
| POK.jpg | cup.jpg | Where is the Pok? |
| NEEM.jpg | Ball.jpg | Where is the Neem? |
| cup.jpg | POK.jpg | Find the Pok! |
| POK.jpg | Ball.jpg | Where is the Pok? |
| Ball.jpg | NEEM.jpg | Find the Neem! |
| cup.jpg | NEEM.jpg | Where is the Neem? |
| Ball.jpg | POK.jpg | Find the Pok! |
| NEEM.jpg | cup.jpg | Find the Neem! |
| LIF.jpg | Shoe.jpg | Where is the Lif? |
| WUG.jpg | car.jpg | Find the Wug! |
| LIF.jpg | car.jpg | Find the Lif! |
| Shoe.jpg | LIF.jpg | Where is the Lif? |
| car.jpg | WUG.jpg | Find the Wug! |
| WUG.jpg | Shoe.jpg | Where is the Wug? |
| car.jpg | LIF.jpg | Find the Lif! |
| Shoe.jpg | WUG.jpg | Where is the Wug? |
| POK.jpg | Shoe.jpg | Where is the Pok? |
| car.jpg | NEEM.jpg | Where is the Neem? |
| LIF.jpg | Ball.jpg | Where is the Lif? |
| WUG.jpg | cup.jpg | Find the Wug! |
| car.jpg | POK.jpg | Find the Pok! |
| NEEM.jpg | Shoe.jpg | Find the Neem! |
| Ball.jpg | LIF.jpg | Find the Lif! |
| cup.jpg | WUG.jpg | Where is the Wug? |

**Supplementary Table 2. Cross Situational Word Learning (CSWL) word learning trials’ list. Stimuli objects are represented in capital letters.**

| BLOCK 1 | | |  | BLOCK 3 | | |
| --- | --- | --- | --- | --- | --- | --- |
| Picture 1 | Picture 2 | Sound |  | Picture 1 | Picture 2 | Sound |
| POK.jpg | dand.jpg | pok-dand |  | LIF.jpg | dand.jpg | lif-dand |
| bink.jpg | POK.jpg | bink-pok |  | bink.jpg | LIF.jpg | bink-lif |
| drit.jpg | POK.jpg | pok-drit |  | drit.jpg | LIF.jpg | lif-drit |
| attention getter | | |  | attention getter | | |
| POK.jpg | bem.jpg | pok-bem |  | bem.jpg | LIF.jpg | bem-lif |
| POK.jpg | doff.jpg | doff-pok |  | doff.jpg | LIF.jpg | lif-doff |
| posk.jpg | POK.jpg | posk-pok |  | LIF.jpg | posk.jpg | lif-posk |
| attention getter | | |  | attention getter | | |
| BLOCK 2 | | |  | **BLOCK 4** | | |
| Picture 1 | **Picture 2** | **Sound** |  | **Picture 1** | **Picture 2** | **Sound** |
| dand.jpg | NEEM.jpg | dand-neem |  | dand.jpg | WUG.jpg | dand-wug |
| NEEM.jpg | bink.jpg | bink-neem |  | WUG.jpg | bink.jpg | wug-bink |
| drit.jpg | NEEM.jpg | neem-drit |  | drit.jpg | WUG.jpg | wug-drit |
| attention getter | | |  | attention getter | | |
| bem.jpg | NEEM.jpg | neem-bem |  | WUG.jpg | bem.jpg | wug-bem |
| doff.jpg | NEEM.jpg | doff-neem |  | WUG.jpg | doff.jpg | doff-wug |
| NEEM.jpg | posk.jpg | neem-posk |  | posk.jpg | WUG.jpg | posk-wug |
| attention getter | | |  | attention getter | | |

**Supplementary Table 3. The slides used in the eBook paradigm. Stimuli words are represented in capital letters.**

| Slide | Narration |
| --- | --- |
| 1 | Title slide: This is a story called Sharing at School! |
| 2 | Tom was getting ready for school and mum told him to take his WUG. |
| 3 | It’s time to go! Tom grabbed his bag but forgot his WUG |
| 4 | When Tom got to school he saw Milly holding her LIF. |
| 5 | Oh no! Tom realized he forgot his WUG and was very sad! |
| 6 | Milly let Tom play with her LIF and that cheered him up! |
| 7 | Tom thanked Milly and off he went to play with the LIF! |
| 8 | At lunch Tom borrowed a POK from his teacher. |
| 9 | He was excited and went to show Milly the POK. |
| 10 | Milly was playing with a NEEM, she was having fun! |
| 11 | Tom had the POK for too long and didn’t want it anymore! |
| 12 | Tom gently asked Milly to trade his toy for the NEEM. |
| 13 | Milly was happy to trade her NEEM and gave it Tom |
| 14 | Closing slide: Tom and Milly were very good at sharing! Great job guys! |

**Supplementary Table 4. Retention test trial list used for the three word learning paradigms. Stimuli objects are represented in capital letters.**

| Picture 1  Top-left | Picture 2  Top-right | Picture 3  Bottom-left | Picture 4  Bottom- right | Sound |
| --- | --- | --- | --- | --- |
| POK.jpg | NEEM.jpg | LIF.jpg | WUG.jpg | Where is the Neem? |
| NEEM.jpg | POK.jpg | WUG.jpg | LIF.jpg | Find the Wug! |
| LIF.jpg | WUG.jpg | POK.jpg | NEEM.jpg | Where is the Lif? |
| WUG.jpg | LIF.jpg | NEEM.jpg | POK.jpg | Find the Pok! |
| NEEM.jpg | LIF.jpg | POK.jpg | WUG.jpg | Where is the Lif? |
| POK.jpg | WUG.jpg | NEEM.jpg | LIF.jpg | Find the Neem! |
| WUG.jpg | LIF.jpg | NEEM.jpg | POK.jpg | Where is the Pok? |
| LIF.jpg | WUG.jpg | POK.jpg | NEEM.jpg | Where is the Wug? |
